# Control of Buckling of Colloidal Supraparticles

**DOI:** 10.1002/smll.202411772

**Published:** 2025-05-02

**Authors:** Lukas J. Roemling, Gaia De Angelis, Annika Mauch, Esther Amstad, Nicolas Vogel

**Affiliations:** ^1^ Friedrich‐Alexander‐Universität Erlangen‐Nürnberg Institute of Interfaces and Particle Technology 91058 Erlangen Germany; ^2^ Soft Materials Laboratory École Polytechnique Fédérale de Lausanne (EPFL) Lausanne 1015 Switzerland

**Keywords:** buckling, colloids, emulsions, self‐assembly, supraparticles, surfactants

## Abstract

The properties of clusters of colloidal particles, often termed supraparticles, are determined by the arrangement of the primary particles. Therefore, controlling the structure formation process is of key importance. While buckled morphologies can result from fast drying kinetics as found in spray drying, controlling the morphology under slow drying conditions remains a challenge. The final morphology of a supraparticle formed from an emulsion droplet can be controlled by manipulating particle–surfactant interactions. Water/oil emulsions are used to template supraparticle formation. The interactions of negatively charged colloidal particles with the surfactants stabilizing the water/oil‐interface are tailored via the local pH within the aqueous droplet. At low pH, protonation of the anionic headgroup of the surfactant decreases electrostatic repulsion of the particles, facilitates interfacial adsorption, and subsequently causes buckling. The local pH of the aqueous droplet phase continuously changes during the assembly process. The supraparticle formation pathway can therefore be controlled by determining the point in time at which interfacial adsorption is enabled by adjusting the initial pH. Consequently, the final supraparticle morphology can be tailored at will, from fully buckled structures, via undulated surface morphologies to spherically rough and spherically smooth supraparticles and crystalline colloidal clusters.

## Graphical abstract

During the confined colloidal self‐assembly in emulsion droplets, the morphology of the resulting supraparticles is determined by particle–surfactant interactions. These interactions can be manipulated via the pH of the aqueous emulsion droplets. The change of pH in the course of the assembly process allows controlling these interactions in time and provides a simple means to tailor the final supraparticle morphology, from fully buckled structures to spherical supraparticles.

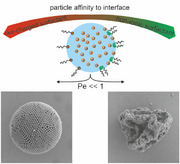

## 1 Introduction

Colloidal particles are useful model systems to fundamentally study self‐organization phenomena^[^ ^1^, ^2^, ^3^ ^]^ and provide functional materials with intriguing electronic,^[^ ^4^, ^5^, ^6^ ^]^ optical,^[^ ^7^, ^8^ ^]^ or magnetic properties.^[^ ^9^, ^10^ ^]^ When such particles are arranged as colloidal crystals, entirely new properties emerge from the collective behavior of those particles. For example, the constructive interference of light scattered at the constituent colloidal building blocks can result in structural coloration.^[^ ^11^, ^12^, ^13^, ^14^, ^15^ ^]^

Supraparticles are interesting in this respect, as such emergent properties can be translated into well‐defined, micron‐scale building blocks that can be dispersed or used as a powder.^[^ ^16^, ^17^, ^18^, ^19^ ^]^ Supraparticles thus combine properties of individual building blocks with novel functionalities enabled by colocalization, defined arrangement, or coupling between these individual building blocks.^[^ ^16^, ^18^, ^20^ ^]^ Since these emergent properties are directly related to the internal structure of such supraparticles, understanding and controlling the formation process is of general importance to reliably tailor their properties.

Supraparticles form by the self‐assembly of colloidal primary particles during the drying of particle‐laden emulsion droplets. These droplets can be produced with different techniques. Spray drying is a fast and scalable technique that produces kinetically controlled structures.^[^ ^21^ ^]^ The drying velocity of droplets on superhydrophobic surfaces can be regulated by the humidity but the process is limited to comparably large supraparticles.^[^ ^22^, ^23^ ^]^ Emulsion templating allows the creation of much smaller droplets, and, with the aid of microfluidic devices, well‐defined supraparticles with narrow size distributions can be produced.^[^ ^24^, ^25^ ^]^ In this system, the diffusion of water through the outer continuous phase is required to consolidate the supraparticles,^[^ ^26^, ^27^, ^28^ ^]^ which enables very slow drying and the formation of highly crystalline clusters.^[^ ^29^, ^30^ ^]^

**Figure 1:**
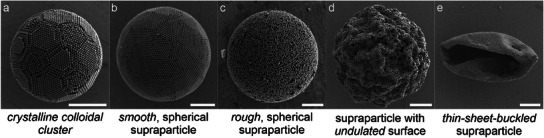
Supraparticles formed by the confined self‐assembly of colloidal primary particles can show different morphologies. a) Defined colloidal clusters with high degree of crystallinity and symmetry (a cluster with icosahedral symmetry is shown). b) Smooth, spherical supraparticle with well‐ordered surface structure. c) Spherical supraparticle with a rough and disordered surface. d) Supraparticle displaying an undulated surface. e) Thin‐sheet buckled supraparticle. All scale bars are 3 µm.

Supraparticles from dried emulsion droplets can exhibit different structures and morphologies (**Figure** **1**). Spherical supraparticles form when the primary particles are free to diffuse and consolidate within the confining droplet. By slowing down the self‐assembly process, the resultant structure can resemble predicted minimum‐energy structures^[^ ^27^, ^29^, ^30^, ^31^ ^]^ with high structural order and defined symmetry such as the icosahedral colloidal cluster shown in Figure 1a. Faster drying conditions typically produce spherical supraparticles with a well‐ordered surface structure (Figure 1b).^[^ ^32^ ^]^ Insufficiently stabilized primary particle dispersions often produce rough supraparticles without long‐range order and random agglomeration of particles (Figure 1c).^[^ ^33^ ^]^ However, a deviation from the spherical shape is also possible if buckling occurs during the drying process. We differentiate between rather spherical supraparticles with an undulated surface (Figure 1d) and the more extreme case of fully collapsed, hollowed thin‐sheets of colloidal particles, resembling a colloidosome (Figure 1e).^[^ ^34^ ^]^ Depending on the application, fully consolidated clusters with a defined internal structure, or buckled supraparticles with increased surface area may be desirable.

Buckling generally describes the collapse of an instable, thin shell. In colloidal‐based materials, buckling is thus associated with the ability of colloidal particles to adsorb and accumulate at liquid interfaces to form a thin shell.

Under fast drying conditions with large Péclet numbers (Pe ≫ 1), the movement of the interface is faster than the diffusion of particles within the droplet. Thus, particles naturally accumulate at the interface to form a thin shell.^[^ ^35^, ^36^ ^]^ This shell eventually becomes sufficiently dense to prevent further consolidation of the particles in the droplet upon drying. Therefore, in such kinetically dominated systems, buckling is often observed.^[^ ^37^ ^]^

However, in emulsion‐based systems where the droplet shrinkage is slow (i.e., Pe ≪ 1), these kinetic effects cannot be observed. Therefore, under such conditions, buckling phenomena need to be associated with the thermodynamically favored adsorption of particles to the liquid interface. This adsorption is a function of the contact angle the particles assume at the interface^[^ ^38^, ^39^ ^]^ and thus relates to the relative affinity of the particles to both phases of an emulsion.^[^ ^40^, ^41^ ^]^ The adhesion strength can vastly exceed k_B_T, in effect causing irreversible particle adsorption.^[^ ^38^, ^42^ ^]^ This interfacial affinity can be exploited, e.g., for the design of Pickering emulsions,^[^ ^43^, ^44^, ^45^ ^]^ colloidosomes,^[^ ^34^ ^]^ particle‐stabilized foams,^[^ ^46^, ^47^, ^48^, ^49^, ^50^ ^]^ or liquid marbles.^[^ ^51^, ^52^ ^]^ In these cases, the particles are often specifically designed^[^ ^43^ ^]^ or surface‐modified^[^ ^53^, ^54^ ^]^ to enable efficient interface adsorption.

During the formation of supraparticles within emulsion droplets, any attraction of the primary particles to the oil/water interface is likely to cause irreversible adsorption, producing an elastic thin shell of particles at the interface.^[^ ^55^ ^]^ Such thin shells subsequently prevent the further consolidation of the primary particles into spherical supraparticles during drying. Ultimately, the collapse of the thin shell thus results in dented or toroidal morphologies^[^ ^56^, ^57^, ^58^, ^59^ ^]^ or more generally in buckled structures.

In droplets produced in conventional microfluidic systems, the continuous phase typically consists of fluorinated oils and Krytox‐based surfactants. ^[^ ^27^, ^31^ ^]^ Most colloidal particles have a low affinity to fluorinated oils, making their adsorption at the drop interface energetically less favorable.^[^ ^38^ ^]^ Nevertheless, buckled structures are still observed in such systems (Figure 1).^[^ ^27^ ^]^ This observation lets us hypothesize that additional mechanisms must be involved to manipulate the interactions between primary particles and the interface of the emulsion.

Here, we focus on the role of surfactants that are generally present in such emulsions to prevent droplet coalescence. However, their role in the self‐assembly process of colloids has been largely overlooked. We elucidate how the particle–surfactant interactions control the affinity of the dispersed particles towards the droplet interfaces. In particular, we utilize a pH‐responsive surfactant with a carboxylic acid end group that can be protonated at low pH values to actively manipulate these interactions. Using charge‐stabilized particles, we adjust the electrostatic repulsion of the particles via pH changes to control the particle affinity to the interface. Thereby, we exert control over the formation pathway of supraparticles and thus control their morphology.

## 2 Results and Discussion

We form emulsion droplets of an aqueous dispersion of negatively charged polystyrene (PS) particles in a continuous fluorinated oil (HFE 7500, 3 M) phase by droplet‐based microfluidics. As surfactants, we compare two widely used fluorosurfactants in such systems.^[^ ^60^, ^61^, ^62^, ^63^, ^64^ ^]^ The anionic surfactant Krytox FSH (the most commonly used and commercially available fluorosurfactant (DuPont)) consists of a perfluorinated polyether (PFPE) with a carboxylic acid head group, and is compared to a nominally non‐ionic surfactant – a triblock of PFPE–O,O′‐Bis(2‐aminopropyl) polypropylene glycol‐block‐polyethylene glycol‐block‐polypropylene glycol (Jeffamine ED‐900)–PFPE (in the following abbreviated as PJP for PFPE–Jeffamine–PFPE). PJP is also commercially available (CreativePEG works) or can alternatively be synthesized through an amide bond formation between Krytox FSH and Jeffamine to form the PJP block copolymer.^[^ ^64^, ^65^, ^66^ ^]^

**Figure 2:**
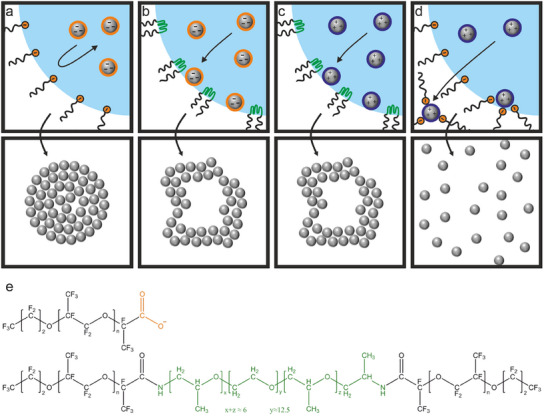
Hypothesis of supraparticle formation pathways as a function of surfactant–particle interactions. a) Negatively charged particles in a droplet stabilized with an anionic surfactant should yield spherical supraparticles. b,c)Non‐ionic surfactant and either negatively (b) or positively (c) charged particles should produce buckled structures after the particles adsorb to the w/o interface. d) Positively charged particles should be attracted by negatively charged surfactant molecules, change their surface functionality, and directly disperse in the outer phase. The chemical structures of the two surfactants at neutral pH are shown in e).

We hypothesize that the interaction of the surfactant with the dispersed particles determines the pathway of the supraparticle formation since the consolidation of the particles happens in the diffusion‐dominated regime (Pe ≪ 1), as illustrated in **Figure** **2**. For the combination of negatively charged particles and negatively charged Krytox FSH, we anticipate that the electrostatic repulsion effectively prevents interfacial adsorption, so that spherical, consolidated supraparticles can be formed (Figure 2a).

When the non‐ionic PJP surfactant is used, no electrostatic interactions should be present to repel the particles and prevent interfacial adsorption. Literature reports even suggest an affinity of the more hydrophobic polypropylene block to hydrophobic polystyrene moieties present at the PS particle surface.^[^ ^67^ ^]^ Therefore, we hypothesize that the PS particles can adsorb to the liquid interface and thus produce buckled structures in the presence of PJP surfactants (Figure 2b). The same result is expected when we invert the particle charge and use positively charged particles in combination with the PJP surfactant (Figure 2c). Finally, the combination of the negatively charged Krytox FSH surfactant with positively charged particles should result in a strong particle–surfactant affinity that may bind the surfactant to the particle surface. We expect such Krytox surface‐modified particles to leave the aqueous droplet as individually dispersed particles into the fluorinated oil, inhibiting the formation of supraparticles (Figure 2d).

In all experiments of this study, we use 1 wt.% of either positively or negatively charged particles and 0.1 wt.% of the commercial anionic surfactant Krytox FSH or the commercial non‐ionic PJP (Figure 2e), unless denoted otherwise. The surfactant concentration is the lowest possible concentration to reliably produce stable emulsions, and thus chosen to minimize interference of excess surfactant during characterization. Spherical supraparticles indeed form when using particles and surfactants with the same charge. The effective prevention of interfacial adsorption is evidenced by the consolidated, spherical particles as the one shown in Figure 1b (see Figure S1 (Supporting Information) for a statistical evaluation) and the well‐defined colloidal cluster in Figure 1a (prepared with reduced droplet shrinkage rate).^[^ ^27^ ^]^ These results agree with different reports in literature on this system.^[^ ^27^, ^62^, ^68^ ^]^

**Figure 3:**
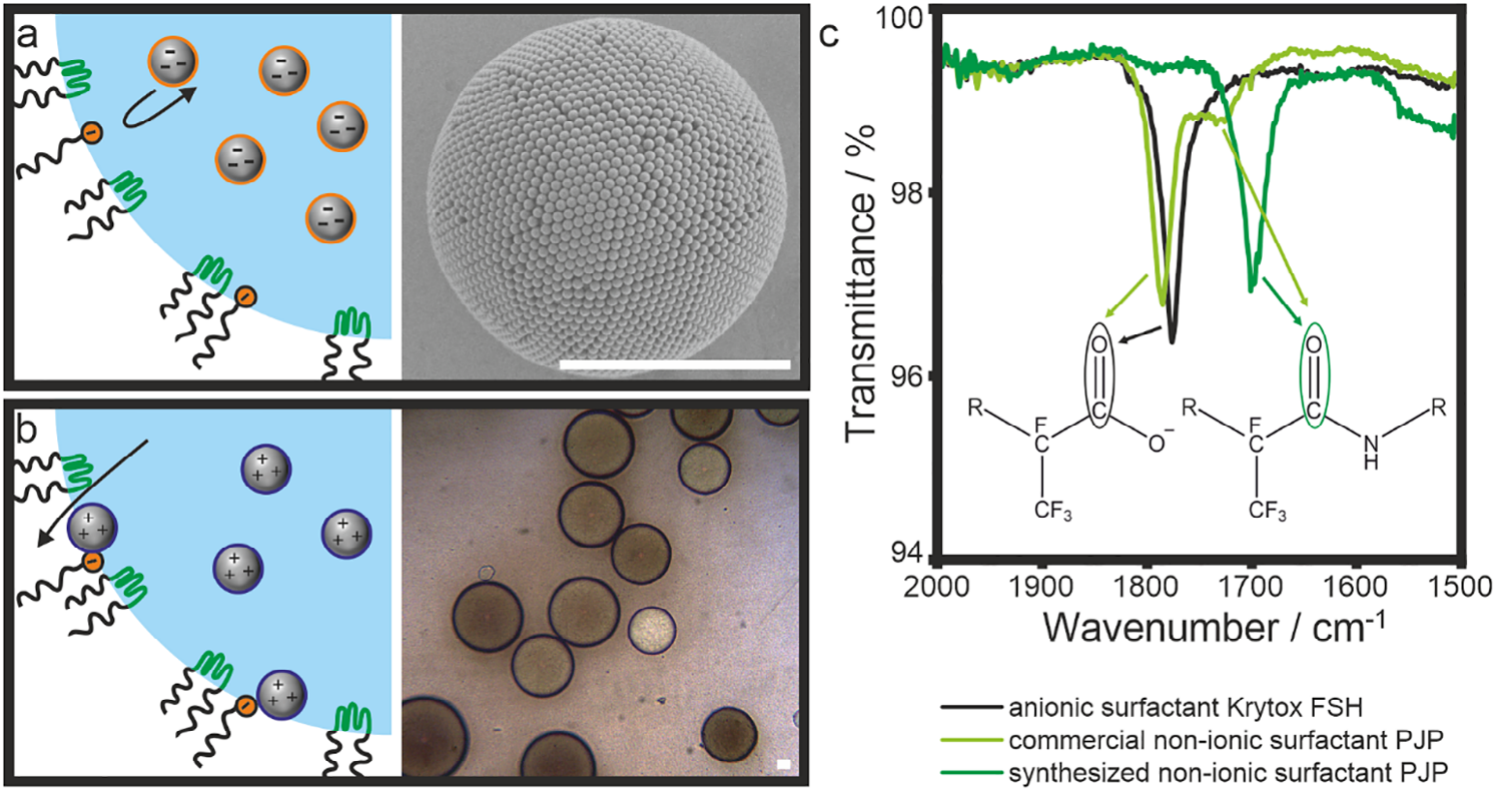
Supraparticles formed within emulsion drops stabilized with the non‐ionic commercial surfactant PJP and characterization of the different surfactants. a) The combination of PJP with negatively charged particles produces spherical supraparticles. b) The combination of PJP with positively charged particles induces individual primary particles to leave the droplet to be dispersed in the continuous phase. c) FTIR spectra of the three surfactants, showing the carbonyl absorption band associated with carboxylic acids, and amides, respectively. All scale bars are 5 µm.

Using oppositely charged particles and surfactants results in the dispersion of primary particles in the outer fluorinated oil phase, as evidenced by a turbid continuous phase after a few minutes of mixing (Figure S2, Supporting Information). This behavior supports our hypothesis of an attractive particle–surfactant interaction that causes the dispersion of individual primary particles within the continuous oil phase (Figure 2d). To our surprise, however, emulsions formed with the non‐ionic PJP surfactants do not show the expected buckling behavior. Instead, the results resemble the samples prepared with Krytox FSH as anionic surfactant (**Figure** **3**; Figures S1,S2, Supporting Information). Combinations of PJP with negatively charged particles produce spherical supraparticles (Figure 3a), while positively charged particles leave the droplet and are dispersed as individual particles (Figure 3b).

This behavior suggests that the non‐ionic surfactant is impure and contains traces of anionic surfactant from the synthesis. First, we measure the interfacial tension via the pendant drop method to investigate the ability of the two surfactants to adsorb to the interface (Figure S3, Supporting Information). The decrease in interfacial tension from 31 ± 1 mN m^−1^ (using 0.1 wt.% Krytox FSH in HFE7500/water) to 18 ± 1 mN m^−1^ (using 0.1 wt.% PJP in HFE 7500/water) demonstrates the superior efficiency of PJP in stabilizing the liquid interface, caused by the increased size of the hydrophilic part of the block‐copolymer. However, a chemical analysis of the commercial PJP surfactant by Fourier transform infrared (FTIR) spectroscopy (Figure 3c) shows the presence of a considerable amount of Krytox FSH residues, the starting material for the synthesis of the block copolymer.^[^ ^69^ ^]^ The presence of free Krytox FSH in the commercial PJP block copolymer can be seen from the C═O stretch vibration of the carboxylic acid group. For pure Krytox FSH, this vibration occurs at ≈1775 cm^−1^ (Figure 3d, black curve). After amide coupling to Jeffamine to synthesize the non‐ionic triblock, the energy of the amide‐based C═O vibration shifts to ≈1700 cm^−1^.^[^ ^70^ ^]^ This new peak is pronounced for a self‐synthesized PJP surfactant (Figure 3c, dark green curve) we prepared following literature protocols.^[^ ^64^ ^]^ In this case, only a minor carbonyl band associated with the carboxylic acid‐based starting material is observed, indicating only small amounts of Krytox FSH impurities. For the commercial PJP, in contrast, the spectral signature of the amide bond only shows up as a shoulder, while the main peak occurs at the spectral position of the Krytox FSH starting material. Hence, we conclude that PJP surfactants can generally contain anionic components of Krytox FSH in varying quantities. This interpretation is further supported by ^19^F NMR shown in Figure S4 (Supporting Information).^[^ ^64^ ^]^ This chemical characterization rationalizes the behavior of the particles within the dispersion droplets in Figure 3. The presence of anionic Krytox FSH facilitates consolidation into spherical supraparticles by repulsive interaction with negatively charged particles (Figure 3a) but cannot confine positively charged particles within the droplet (Figure 3b). Note that the behavior of the synthesized surfactant with presumably higher purity was similar in the same emulsion system (Figure S5, Supporting Information).

Therefore, to investigate the behavior of the pure non‐ionic surfactant, and, in particular, to probe if the reduced repulsion causes the formation of buckled supraparticles requires a different approach. We capitalize on the pH‐dependent surfactant properties of carboxylic acid‐containing Krytox FSH. If the pH of the inner water phase is below the pK_A_ value of the carboxylic acid headgroup, protonation of the headgroup will eliminate the anionic charge, thus removing the anionic impurities in the non‐ionic surfactant and reducing the impact of these impurities on the structure formation process.

The pK_A_ values for carboxylic acids with different chemical environments are reported to range from 2 to 4.^[^ ^71^ ^]^ The interfacial tension between a fluorinated oil droplet containing 0.1 wt.% Krytox FSH and water shows a significant increase if the pH of the aqueous phase is shifted from 5 to 3 (Figure S6, Supporting Information), indicative of the reduced hydrophilicity of the headgroup. This trend is not observed for the commercial PJP surfactant, indicating that in this case, the non‐ionic block copolymer remains interfacially active at all pH values (Figure S6, Supporting Information). As even small amounts of surfactant impurities can significantly change the assembly process (Figure 3a,b), we anticipate that adjusting the pH will enable us to control the particle–surfactant interactions in our system by manipulating electrostatic repulsion, while maintaining a stable emulsion droplet by the interfacial activity of the non‐charged block copolymer surfactant.

**Figure 4:**
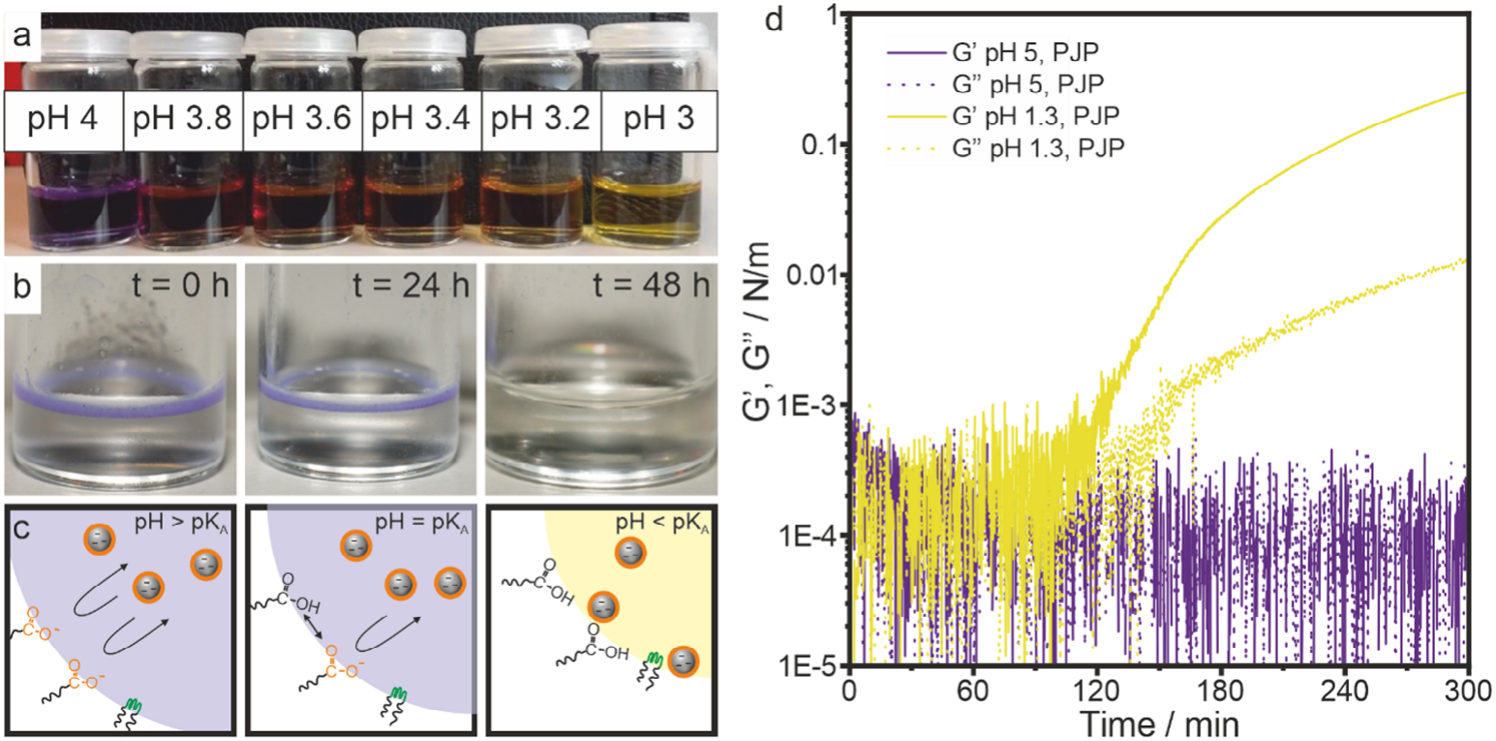
The local pH changes within emulsion droplets upon shrinkage in water/fluorinated oil emulsions, affecting the affinity of particles to the w/o interface. a) Aqueous solutions of bromophenol blue show a color change from violet/blue to yellow between pH 4 and 3. b) Evolution of the pH inside w/o emulsion droplets over time, shown by the color change of bromophenol blue. c) Schematic illustration of the protonation of the PJP surfactant at the water‐oil interface during drying, leading to an increased affinity of the particles to the interface. d) Interfacial rheology shows that the storage (G′, solid lines) and loss moduli (G″, dotted lines) of an interface between an aqueous particle dispersion and the fluorinated oil phase stabilized by 0.1 wt.% PJP (commercial), increase at pH 1.3 (below the pK_A_ of Krytox FSH) but are constant at pH 5 (above the pK_A_ of Krytox FSH), indicating particle adhesion to the interface at low pH.

Additionally, due to the non‐polar character of the continuous fluorinated oil phase, we do not expect any charged moieties to be able to leave the aqueous phase. Therefore, we hypothesize that the diffusion of water molecules into the oil phase during droplet shrinkage continuously changes the pH within the water droplet of the emulsion. Thus, when starting with an initial moderate concentration of H^+^ ions (i.e., a pH above the pK_A_ value of the carboxylic acid headgroup), the proton concentration will continuously increase, and the pH value correspondently decrease, as water molecules diffuse out of the droplet during the drying process. We test this hypothesis using emulsions of pure water droplets with the pH indicator bromophenol blue in a continuous fluorinated oil phase (**Figure** **4**). Bromophenol blue shows a color change from purple to yellow between pH 4 and pH 2 (Figure 4a). We adjust the initial conditions to pH 4 and the formed emulsions show a purple color (Figure 4b). In the course of the drying process, the color of the emulsion changes to pale yellow (Figure 4b), demonstrating that the local pH within the emulsion droplets indeed changes upon droplet shrinkage.

This ability to manipulate the local pH provides us with a handle to control the point in time at which the surfactant properties of the particle‐laden emulsion droplet (and with it the particle affinity to the interface) changes, as shown in Figure 4c. We expect that at moderate or high pH values (pH > pK_A_ (Krytox FSH)), negatively charged colloidal particles will be repelled from the interface due to the electrostatic repulsion of the deprotonated carboxylic acid headgroup of the surfactant. At low pH, where the carboxylic acid head group is protonated, we expect the particles to adhere to the w/o interface due to a lack of repulsive forces and their affinity to the PEG–PPG–PEG moiety of the non‐ionic surfactant (Figure 4c).^[^ ^67^ ^]^ To support this hypothesis, we use interfacial rheology to investigate the affinity of the charge‐stabilized particles to the surfactant‐covered interface at two different pH values, chosen to be far below the pK_A_ (pH 1.3) and far above the pK_A_ (pH 5) (Figure 4d). At pH 1.3, where nearly all surfactant molecules are uncharged, the 2D storage (G′) and the loss moduli (G″) increase after 90 min, indicating an increased elasticity of the interface, associated with the adsorption of particles.^[^ ^72^ ^]^ At pH 5, where the surfactant is negatively charged, the storage and loss moduli remain constant (at the limit of detection) for the entire experimental time frame of 5 h, indicating that the nature of the interface did not change. We therefore conclude that at high pH values (pH 5), interfacial adsorption is prevented by the electrostatic repulsion between surfactant and particles. Conversely, if the pH is chosen to be below the pK_A_ value of the surfactant, protonation of the carboxylic acid head group removes charges and increases the interfacial affinity of the particles. As a result, a monolayer of particles adsorbs to the oil/water interface.

The color change of the pH indicator demonstrates that the pH inside the emulsion droplets changes upon droplet shrinkage. This change in pH leads to the protonation of the surfactant and thus triggers particle adsorption to the oil/water interface. We now demonstrate that we can leverage these two properties to control the formation pathway, and thus the final morphology of the supraparticles. We adjust the initial pH within droplets of an aqueous colloidal dispersion (1 wt.% negatively charged PS particles) to a defined initial pH by the addition of HCl. The choice of this initial pH determines at which point during the drying process the surfactant will be protonated, and thus if and when interfacial adsorption of particles sets in.

**Figure 5:**
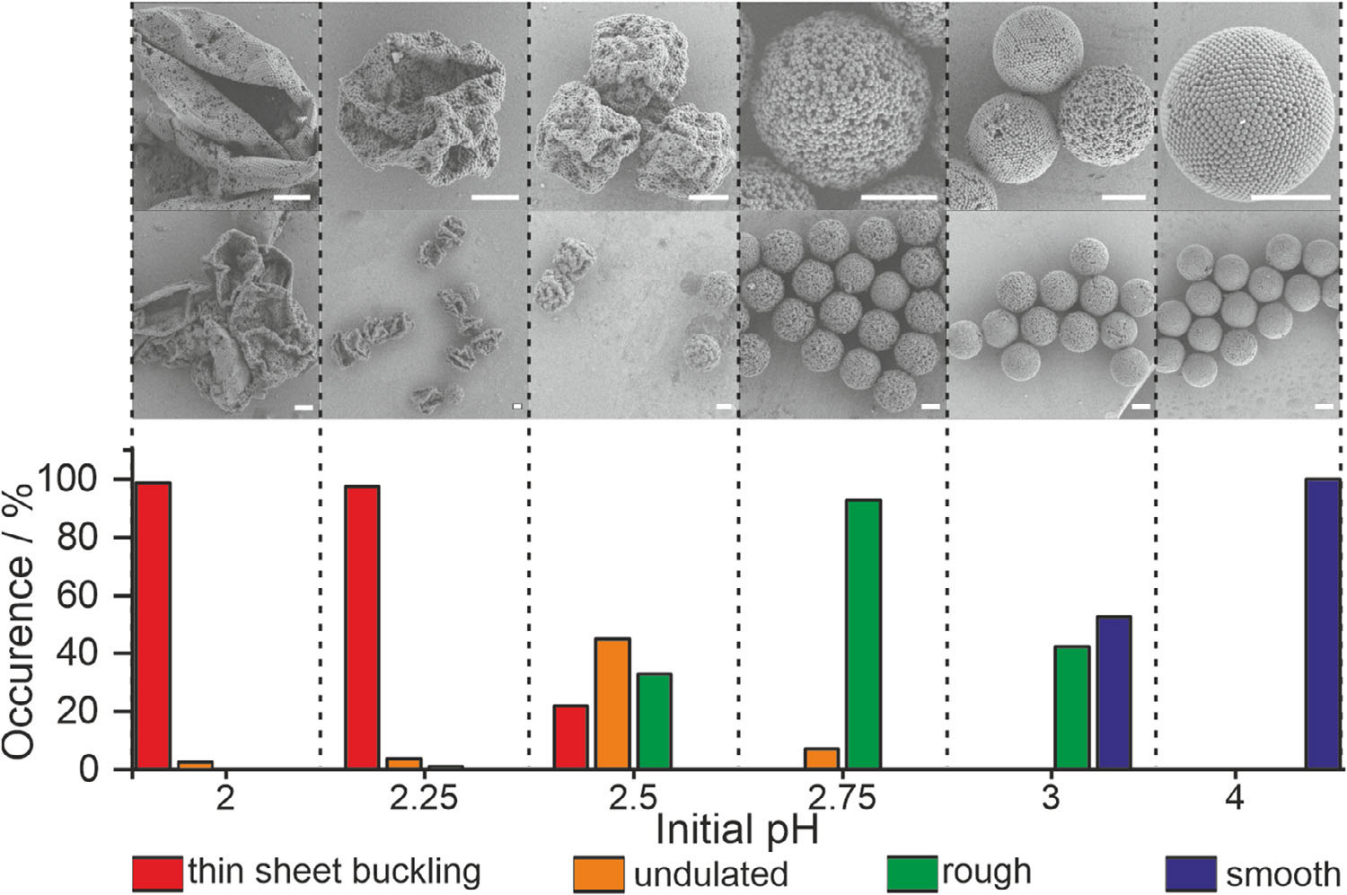
Control of supraparticle morphology is achieved by adjusting the initial pH within the emulsion droplet. The supraparticles are characterized via statistical analysis of SEM images. Sample sizes for each data point can be found in Table S2 (Supporting Information). All scale bars are 3 µm.

We use droplet‐based microfluidics^[^ ^18^ ^]^ to produce uniform emulsion droplets within a continuous fluorinated oil phase containing the commercial PJP surfactant with a concentration of 0.1 wt.%. The supraparticles form by shrinkage of the particle‐laden droplets at room temperature for ≈24–36 h. The resulting morphologies are subsequently characterized using statistical evaluation of Scanning Electron Microscopy (SEM) images (**Figure** **5**). The Péclet number at initial conditions, which gives a ratio of the time needed for consolidation and the characteristic time of diffusion,^[^ ^73^ ^]^ is Pe  =  3.5 × 10^−3^ (assuming conditions for maximal Pe, with the largest droplet size (46 µm) and the fastest drying time (24 h). The complete calculation can be found in Table S1 (Supporting Information)). This underlines that diffusion rather than convection is the dominating mode of particle movement and suggests that kinetic effects, as found, e.g., in shell formation in spray drying,^[^ ^37^, ^58^ ^]^ do not occur in our system.

For the evaluation we distinguish four classes of supraparticle morphologies, as introduced in Figure 1b–e: *smooth* supraparticles with a well‐defined spherical shape and ordered surface structure; *rough* supraparticles with a consolidated spherical shape but no visible order at their surface; *undulated* supraparticles with a near‐spherical shape but pronounced buckled features at the surface; and *thin‐sheet buckled* supraparticles with a surface that is reminiscent of a thin sheet that crumbled under pressure.

As shown in Figure 5, drying of particle‐laden droplets with an initial size of 25 µm and an initial pH value of 2 to 2.25 results in the almost exclusive appearance of thin‐sheet buckled supraparticles. Increasing the initial pH value to 2.5 predominantly produces undulated supraparticles. A further increase in pH to 2.75 results in mostly rough supraparticles. At an initial pH 3, only spherical supraparticles are observed. Their topology is either rough or smooth, with both morphologies occurring in close to equal ratios. Finally, with an initial pH of 4 smooth supraparticles exclusively form. Note that such supraparticles can be crystallized into well‐ordered colloidal clusters with defined geometries, such as the icosahedral cluster shown in Figure 1a, by further reducing the shrinkage rate.^[^ ^27^, ^29^ ^]^

**Figure 6:**
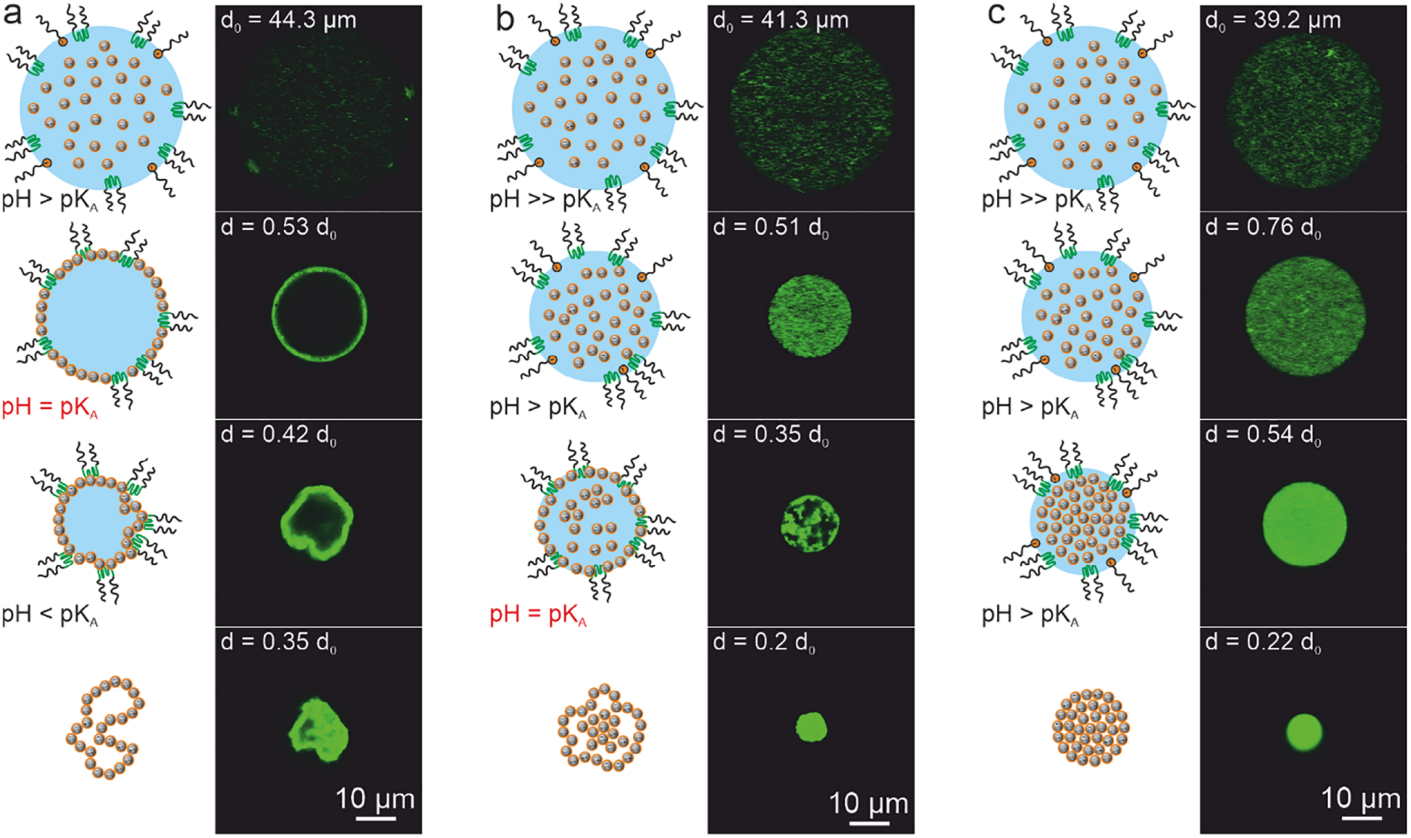
Schematic representation of the drying process of a w/o emulsion droplet containing colloidal particles with varying initial pH conditions and confocal microscopy images of the middle plane of such droplets at different times of the drying process. a) At an initial pH=2, the pKA is reached early in the supraparticle formation process and particles adsorb to the interface and form of a thin shell. Further drying leads to thin‐sheet buckled structures. b) An initial pH value of pH=3 leads to a delayed adsorption of the particles to the interface. As a result, the particles can further consolidate in the bulk volume, which then reduce the degree of buckling and produces supraparticles with undulated surfaces. c) Higher initial pH values (pH=5) suppress particle adsorption to the interface and cause the formation of spherical supraparticles.

We use confocal microscopy to assess the formation pathway as a function of initial pH and thus to investigate when the dispersed particles adsorb to the droplet interface and how this controls the resulting supraparticle morphology (**Figures** **6** and Video S1,S2,S3, Supporting Information). Images were taken of the middle plane of a droplet containing fluorescently labeled particles (Fluoresbrite, d = 200 nm). The particle concentration was reduced to 0.27 wt.% to avoid the saturation of the detector and 3 different initial pH conditions were used (a) pH 2; b) pH 3; and c) pH 5). Drying of the colloidal droplet occurred faster in the confocal setup (fastest drying ≈90 min), which is most likely due to the increase in local temperature caused by the excitation with the laser. However, this process is characterized by a Péclet number of Pe =  3.7 × 10^−2^, and thus is still diffusion controlled (Table S1, Supporting Information).

If the initial pH value is close to the pK_A_ value of the carboxylic acid head group of the surfactant (pH ≤ 2.25), protonation occurs at early stages of the drying process (Figure 6a; Video S1, Supporting Information). Fluorescent images reveal an increase in fluorescence signal at the surface of the emulsion droplet and a reduction of signal intensity in the bulk of the droplet, already at an early stage in the process (Figure S7, Supporting Information). Upon further drying the particle layer is compressed, collapses, and forms a thin‐sheet buckled structure (Figure 6a), similar to the morphology observed in the SEM (Figure 5). The collapse of the thin sheet (rather than an expulsion of the particles) is likely governed by the elastic nature of the shell and the low affinity of the particles to the oil‐phase.^[^ ^41^ ^]^ If the initial pH value is higher than the pK_A_ value of the carboxylic acid group, e.g., for an initial pH = 3 (6b), the particles will start to adsorb to the interface at a later stage of the formation process, when the shrinking droplet reaches the pK_A_ of the surfactant (Figure 6b; Video S2, Supporting Information). In‐situ confocal images show that the adsorption of particles to the droplet surface begins at roughly 42% of the initial diameter d_0_ (≈7.5% of the initial volume) (Figure S8, Supporting Information). This would result in a reduction of the pH to slightly below 2, in agreement with the results for the lower initial pH (Figure 6a). As a result, with the initial pH = 3, buckling will occur when the size of the droplet is already more reduced and the available interfacial area is smaller. Thus, less particles can adsorb to the interface, while more particles remain inside the droplet (Figure 6b, third row), producing undulated supraparticle morphologies after complete consolidation.

Finally, with an initial pH = 5 (Ultrapure water left at ambient conditions), the pH never decreases below the pK_A_ and spherical supraparticles result as the primary particles can consolidate within the spherical confinement without any interference from the liquid interface (Figure 6c; Figure S9, Video S3, Supporting Information), corroborating the statistical evaluation for an initial pH = 4 (Figure 5).

**Figure 7:**
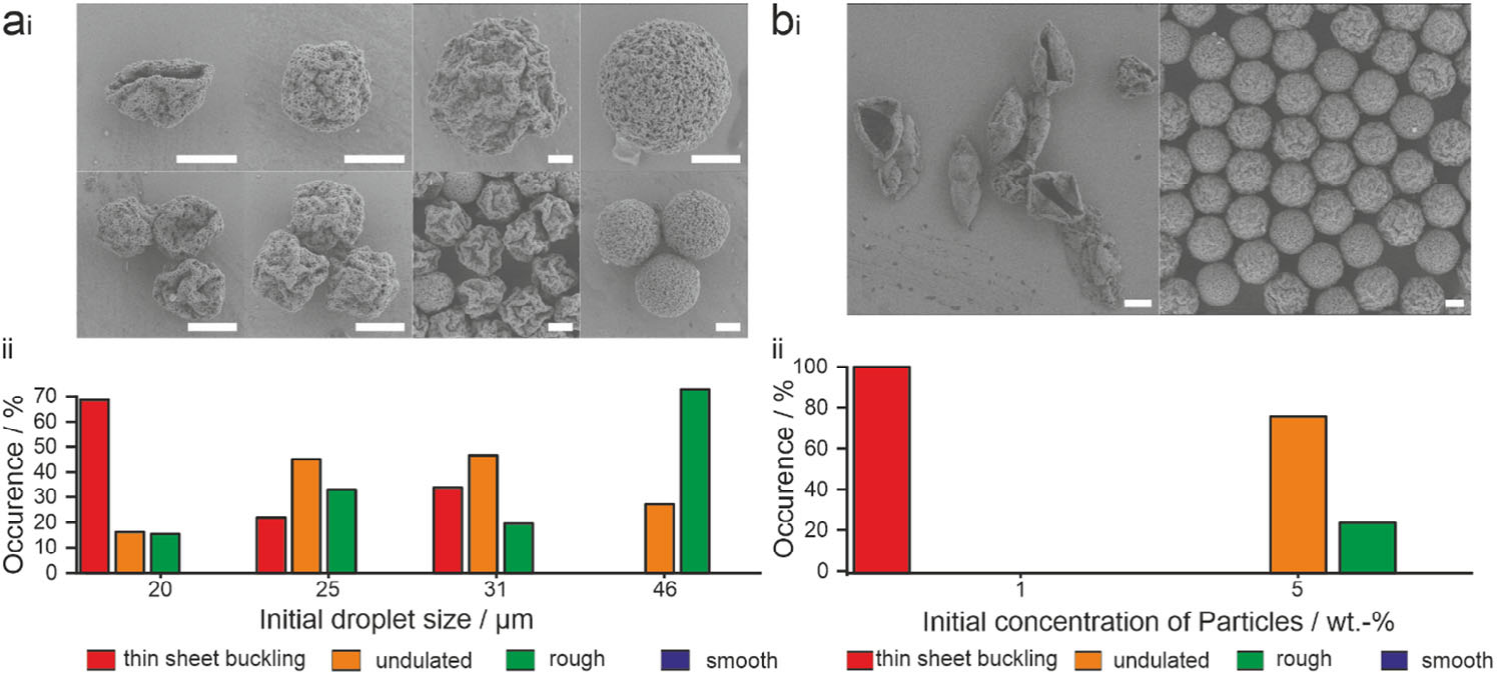
Control of supraparticle morphology via the number of primary particles available in the bulk of the droplet. a) Influence of droplet size. W/O emulsions were produced at an initial pH = 2.5 with different droplet sizes at the same particle concentration (1 wt.%). i) representative SEM images of the formed supraparticles; ii) statistical evaluation of the occurrence of characteristic morphologies. b) Influence of solid content. W/O emulsions were prepared with an initial droplet size of 20 µm, at an initial pH = 2 with different primary particle concentrations. i) representative SEM images of the formed supraparticles; ii) statistical evaluation of the occurrence of characteristic morphologies. All sample sizes can be found in Table S2 (Supporting Information). All scale bars are 5 µm.

Having established the formation mechanism, we can leverage other experimental parameters to control supraparticle morphologies. First, we tune the final morphology via the droplet size. Larger droplets have a lower surface—to–volume ratio than smaller droplets. With conditions favoring particle adsorption to the interface early in the formation process (protonated Krytox FSH surfactant; pH 2.5), the interface will be readily covered with particles, templating buckled morphologies. With increasing droplet size, the surface‐to‐volume ratio decreases, leaving a higher ratio of particles in the bulk when the interface is saturated with particles. The degree of buckling should thus be less pronounced (Figure S10a, Supporting Information). We demonstrate this control by adjusting the initial droplet size, using a fixed primary particle concentration and statistically evaluate the supraparticle morphologies via SEM image analysis as a function of the initial emulsion droplet size (**Figure** **7**a). We control the droplet size in our microfluidic device by using channels with a diameter of 15 or 25 µm. Additionally, we use a low ratio of inner flow rate (water phase) and outer oil phase for the small droplets and a high ratio to obtain larger droplets. The detailed parameters can be found in the Materials and Methods section. We observe a shift from thin‐sheet buckled supraparticles as the main morphology for small initial droplet sizes (20 µm), via predominantly undulated morphologies (25–31 µm initial droplet sizes) to mainly rough supraparticles (46 µm initial droplet size). Note that the supraparticle diameter increases linearly with the increase of the droplet size when measuring supraparticles of the same morphology. We show this correlation in Table S3 (Supporting Information) for the case of the rough, spherical supraparticles.

In a similar fashion, the resultant morphology depends on the initial primary particle concentration, since it determines the number of particles that remain in the bulk liquid after interfacial adsorption. When increasing the particle concentration from 1 to 5 wt.% with constant initial droplet sizes (20 µm) and constant surfactant concentration (0.1 wt.%), the main fraction of observed morphologies shifts from thin‐sheet buckled supraparticles, to undulated and rough supraparticles (Figure 7b; Figure S10b, Supporting Information). While with the low particle concentration, nearly all primary particles adsorb to the liquid interface, and thus produce a fully buckled morphology, the increased number of primary particles retains particles in the bulk and thus decreases the degree of buckling upon consolidation.

## 3 Conclusion

Supraparticles formed by the confined assembly of colloidal primary particles within emulsion droplets can exhibit different morphologies. We identify particle–surfactant interactions as the determining factor to control these morphologies. Efficient repulsion, caused by like‐charged systems produces spherical supraparticles. Insufficient repulsion or weak attraction causes interfacial adsorption of the particles, triggering buckling. Strong attraction, for example by using oppositely charged systems does not produce stable supraparticles as surfactant‐coated primary particles can leave the droplet.

Two key parameters can be generalized to understand and successfully predict buckling in colloidal supraparticle formation. First, if particles do not adhere to the interface, no buckling will be observed. If particles adhere to the interface, the irreversible adsorption causes buckling upon further shrinkage. Second, the point in time at which the particles adsorb to the interface during the formation process and how many particles are left in the bulk of the droplet will determine the resulting morphology.

In well‐established water‐in‐fluorinated oil emulsions stabilized with Krytox based surfactants, we found that non‐ionic fluorinated surfactants contained traces of anionic surfactant from the synthesis. The point in the formation process at which the interfacial adsorption occurs can be controlled via the protonation of the charged surfactant headgroup, triggered by a continuous change in pH within the emulsion droplet. The number of particles left in the bulk can be controlled by adjusting the particle concentration in the droplet, or by changing the surface–to–volume ratio via the droplet size. These insights provide simple handles to reliably control the morphology and thus the properties of supraparticles formed by emulsion templating.

## 4 Experimental Section

### Synthesis of Charge‐Stabilized Polystyrene Colloidal Particles

The synthesis was adopted from literature.^[^ ^74^ ^]^ In brief, 250 mL of water was heated up to a temperature of 80 °C in a three‐necked‐flask equipped with a reflux condenser. The system was constantly flushed with nitrogen. After heating, 10 g of styrene (>99%, Sigma Aldrich) was added. After another 10 min, 0.052 g of sodium 4‐vinylbenzenesulfonate (>90%, Sigma Aldrich), dissolved in 5 mL of water was added as a comonomer and stirred for 5 min before 0.1 g of ammonium persulfate (>98%, Sigma Aldrich) dissolved in 5 mL of water was added. After 22 h, the nitrogen flow and heating were stopped, and the colloid was left to cool. The resulting polymer colloidal dispersion was filtered through a lint‐free tissue, extensively dialyzed and resulted in particles with a diameter of d = 246 ± 2 nm and a zeta potential of ζ = −35 mV.

For positively charged particles, the synthesis protocol shown above was adapted to use (vinylbenzyl)trimethylammonium chloride (VBTMAC, 99% Sigma Aldrich), as a comonomer instead of acrylic acid and 2,2‐azobis(2‐methylpropionamidine) dihydrochloride (AAPH, 97% Sigma Aldrich) as an initiator instead of APS, based on previous publications.^[^ ^75^ ^]^ In brief, in a three‐neck round‐bottom flask with 240 mL water was heated in an oil bath (60 °C oil bath temperature) under constant nitrogen flow and stirring (ellipsoidal magnetic stirrer, VWR). A cooler provided constant reflux of the condensate. After 40 min, 15 g styrene was added, when the reaction temperature was reached. 0.63 g of VBTMAC and 0.28 g of AAPH, dissolved in 5 mL of water respectively, were added in 5 min intervals. The addition of AAPH marked the beginning of the reaction. After 20 h, the heating and nitrogen flow was stopped. After cooling, the formed dispersion was filtered through a lint‐free wipe (Kimberly‐Clark) and stored until further use.

### Synthesis of Non‐Ionic Fluorinated Surfactant

The non‐ionic triblock surfactant of Krytox FSH–Jeffamine900–Krytox FSH was bought from Creative PEGWorks and used as received and compared against the same surfactant synthesized following a protocol from literature.^[^ ^64^ ^]^ For this, 5 g of Krytox FSH (DuPont) were stirred under Argon (Ar) atmosphere. One drop of anhydrous DMF (> 99%, Sigma Aldrich) and 0.8 mL of 2 m oxalyl chloride in DCM (Sigma Aldrich) were added and the mixture was stirred for 4 h. Afterward, the DCM and leftover oxalyl chloride were removed by rotary evaporation (Hei‐VAP, Heidolph, Germany) for 2 h, at 40°and 1 mbar. The product was dissolved in 10 mL Novec 7100 (3M) and set under Ar atmosphere and 0.5m equivalent of dry Jeffamine900 (Sigma Aldrich) dissolved in 5 mL anhydrous DCM (> 99.8%, Sigma Aldrich) was added. The reaction was left to reflux at 65 °C overnight. To purify the surfactant 2 mL of Novec 7100 (3M) and 50 mL of Methanol (> 99 %, Sigma Aldrich) were added to precipitate the block copolymer, which was centrifuged at 3 °C and 3000 RPM (Mega Star, 1.6R, VWR). The supernatant was discarded and the cleaning procedure was repeated four times to afford a pale white product.

### Production of Microfluidic Channels

Sylgard 184 was purchased from Biesterfeld AG. Resin and crosslinker were mixed in a ratio of 10:1, degassed for 10 min under vacuum, and subsequently poured onto a Silicon master. It was cured at 80 °C for 24 h. Afterward, the PDMS was removed from the mold, activated in O_2_‐plasma for 30 s, and bonded on an activated glass slide. The channels were then flushed with 3 wt.% Trichloro(1H,1H,2H,2H‐perfluorooctyl)silane (Sigma Aldrich, 97%) in Novec HFE 7500 (3M) to turn the channels fluorophilic. The solution was left in the channels for 24 h and then stored at 80 °C for another 24 h to ensure functionalization of the channels and complete evaporation of excess HFE 7500.

### Preparation of Glass Vials

1.5 mL glass vials (Carl Roth) were placed in a beaker filled with denatured ethanol (Carl Roth, 96%) and ultrasonicated for 1 min. The glass vials were then dried and activated in O_2_‐plasma for 5 min. Afterward, the vials were placed in a desiccator and 50 µL of Trichloro(1H,1H,2H,2H‐perfluorooctyl)silane was added to the chamber. After storage under vacuum for 24 h the vials were heated to 80 °C for 2 h and rinsed with denatured ethanol to get rid of excess silane.

### Supraparticle Formation

The microfluidic channel consisted of 2 inlets and 1 outlet. The inlet for the inner phase was connected to a syringe pump with the aqueous particle‐laden phase, while the inlet for the outer phase was connected to the outer fluorinated phase, which consisted of 0.1 wt.% PFPE5000–Jeffamine900–PFPE5000 (Creative PEGWorks) in Novec HFE 7500 (3M). The flow velocities could be adjusted separately. The resulting droplet size was adjusted by using two different channel sizes and varying the flow rates of the water/oil phases. The smallest droplet size (20 µm) was obtained using a 15 µm channel and flowrates of 50/800 µL h^−1^, 25 µm droplets from the 15 µm channel and flowrates 50/200 µL h^−1^. 31 µm droplets were produced with the 25 µm channel and 50/200 µL h^−1^ flowrates and 46 µm droplets were produced by using the 25 µm channel and 200/100 µL h^−1^ flowrates. The resulting emulsion was collected in a pipette tip and subsequently transferred to a sample vial with a fluorinated surface to avoid droplet coalescence with the glass surface. The vial was stored without a cap at room temperature to allow water to diffuse out of the droplets and evaporate to induce assembly. The formation process took ≈24–36 h. The setup was schematically shown in Figure S11 (Supporting Information).

### Characterization

The supraparticles were characterized using a GeminiSEM 500 (Carl Zeiss, Germany) and the software ImageJ. Surfactants were characterized using 1) FTIR (Spectrum 3, PerkinElmer); 2) NMR spectroscopy (Bruker AVANCETM‐400 spectrometer operating at 400 MHz. Samples were dissolved at a concentration of 10 mg in 0.75 mL of hexafluorobenzene before analysis); 3) pendant drop measurements (DSA 30 drop shape analyzer, Krüss); and 4) interfacial rheology (DHR‐3, TA Instruments) using a double wall ring (DWR) geometry.

### Confocal Microscopy

Confocal microscopy images were taken with a Leica TCS SP5 II microscope using HCX PL APO 63x/1.20 lens. Fluorescent particles (d = 200 nm, Fluoresbrite) were bought from Polysciences, centrifuged, redispersed in water/HCl mixtures and the droplets were emulsified using microfluidics. The emulsion was injected into a PDMS block with a cavity of 100 µm height 2 × 2 cm width joint to a cover slide. The mold for the PDMS block was prepared using a 3D printer (DecorousViper, formlabs) filled with the resin Tough2000. The mold was designed using TinkerCAD.

### Statistical Analysis

The statistical analysis of the supraparticle morphologies was done using SEM images. For every sample, a minimum of 50 supraparticles were counted and the occurrence of the single morphologies was calculated in percent before being plotted. The total sample size of each parameter combination could be found in Table S2 (Supporting Information).

## Conflict of Interest

The authors declare no conflict of interest.

## Supporting information



Supporting Information

Supplemental Movie 1

Supplemental Movie 1

Supplemental Movie 1

## Data Availability

The raw and metadata used to prepare the manuscript are publicly available in Zenodo at https://zenodo.org/records/15065436.
