# Supplementary material for: Control of Buckling of Colloidal Supraparticles
Source: Small. 2025 May 2;21(22):2411772. doi: 10.1002/smll.202411772 (PMC12138862; doi:10.1002/smll.202411772)
Supplement: Supplementary file 1 — Supporting Information [file SMLL-21-2411772-s003.pdf]

NANO · MICRO
small

Supporting Information

for *Small*, DOI 10.1002/smll.202411772

Control of Buckling of Colloidal Supraparticles

*Lukas J. Roemling, Gaia De Angelis, Annika Mauch, Esther Amstad and Nicolas Vogel**

Supporting Information

Control of Buckling of Colloidal Supraparticles

*Lukas J. Roemling, Gaia De Angelis, Annika Mauch, Esther Amstad, Nicolas Vogel**

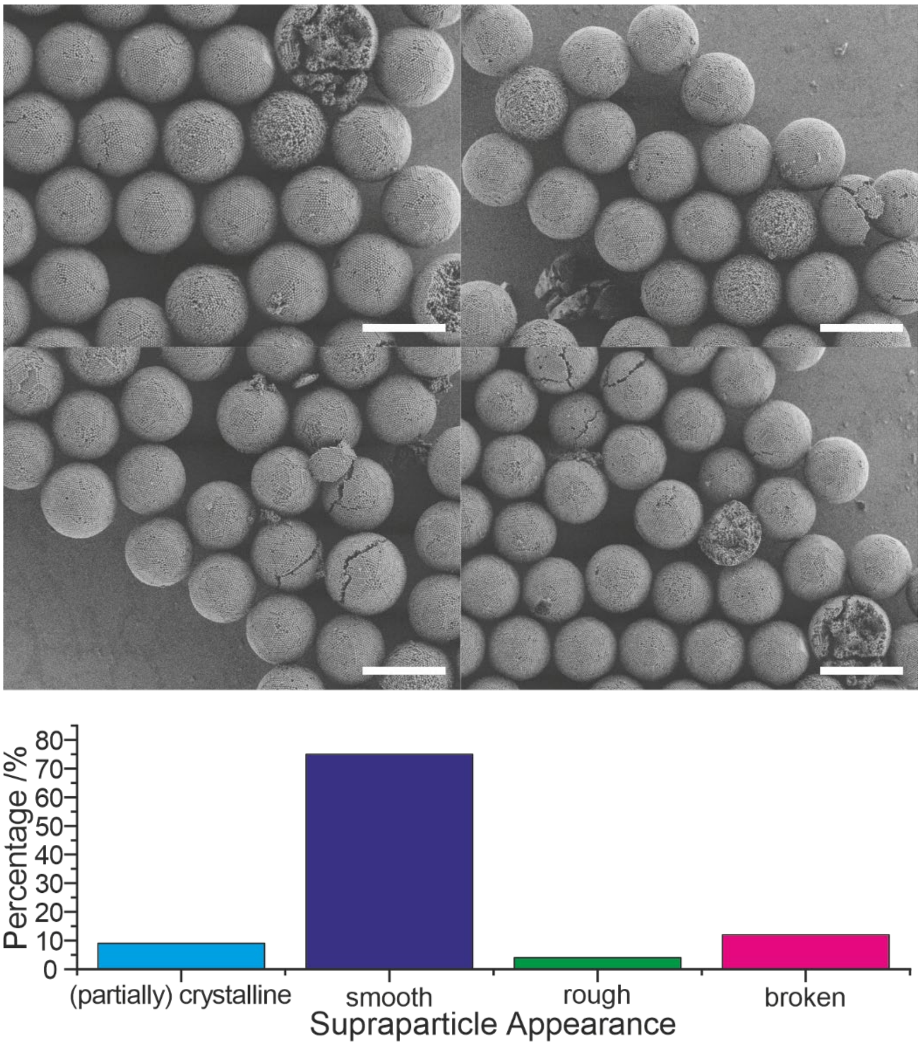

Figure S1. Low-resolution SEM images of supraparticles produced via microfluidics using negatively charged polystyrene primary particles and anionic Krytox FSH surfactant. 75% of the cluster appeared to have a smooth surface, 9% were partially crystalline clusters, 12 % of the supraparticles were broken, probably due to the deposition (after the consolidation of the supraparticle). Only 4% of the supraparticles showed a rough surface without apparent order.

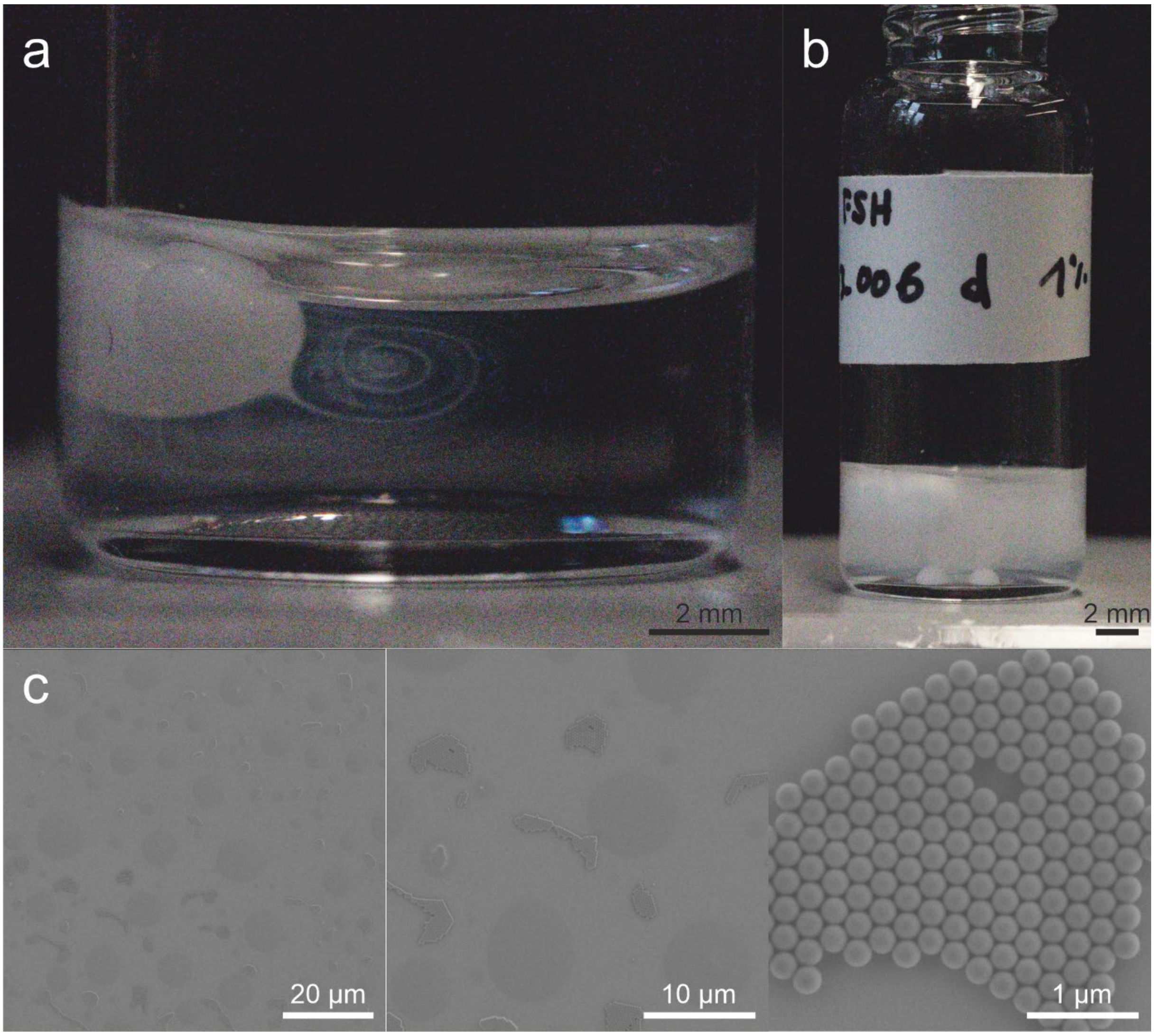

Figure S2. Photographs and SEM images of water-in-oil emulsion with positively charged particles in the water droplet. The oil phase contains anionic Krytox FSH. (a) Shortly after emulsification the continuous outer oil phase becomes turbid due to particles dispersing in the fluorinated oil. (b) After several minutes, the entire oil phase starts to turn opaque. (c) SEM images of substrates, where a drop of the turbid oil was deposited. They show patches of particles distributed over the substrate (The spherical dark spots result from the excess surfactant while the non-spherical spots are small arrangements of particles). These hexagonally ordered patches (see high magnification image on the right) are reminiscent of SEM images of aqueous particle dispersions dried on a substrate, indicating that the particles are dispersed as single particles in the oil-phase after several minutes.

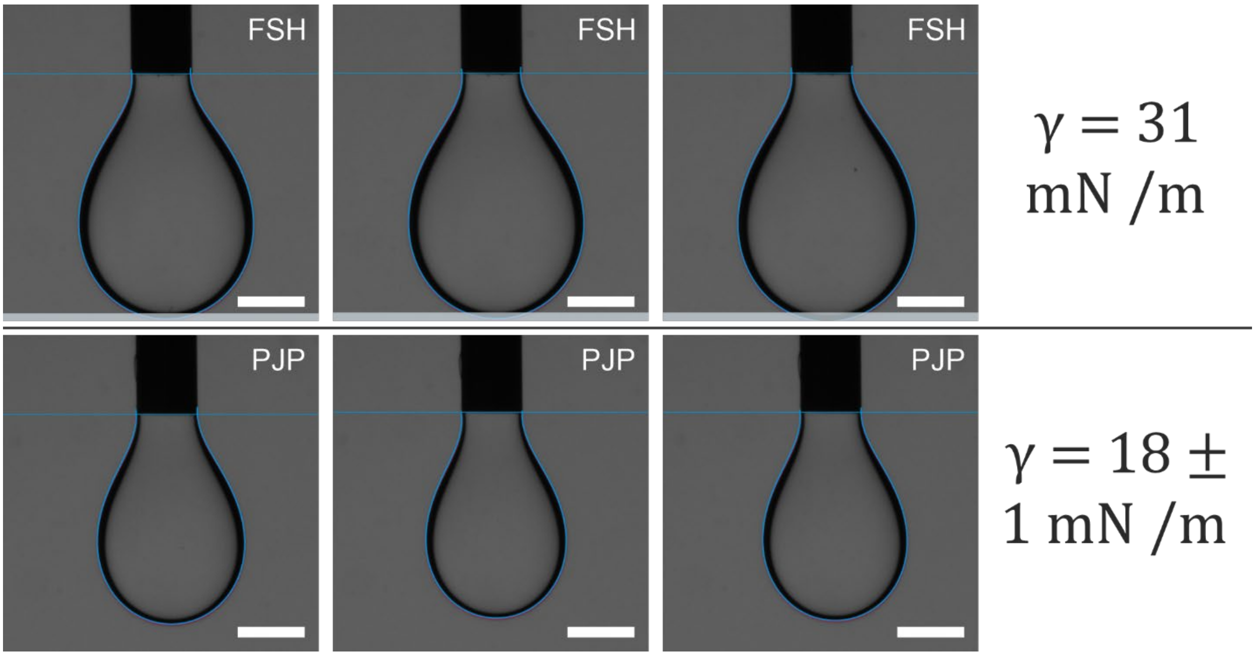

Figure S3. Surface tensions of droplets of fluorinated oil (HFE 7500) in water with 0.1 wt.-% of different surfactants (top: Krytox FSH; bottom: PJP triblock-copolymer) measured using the pendant drop method. PJP reduces the interfacial tension between the phases considerably more than FSH. Scale bars are 1 mm.

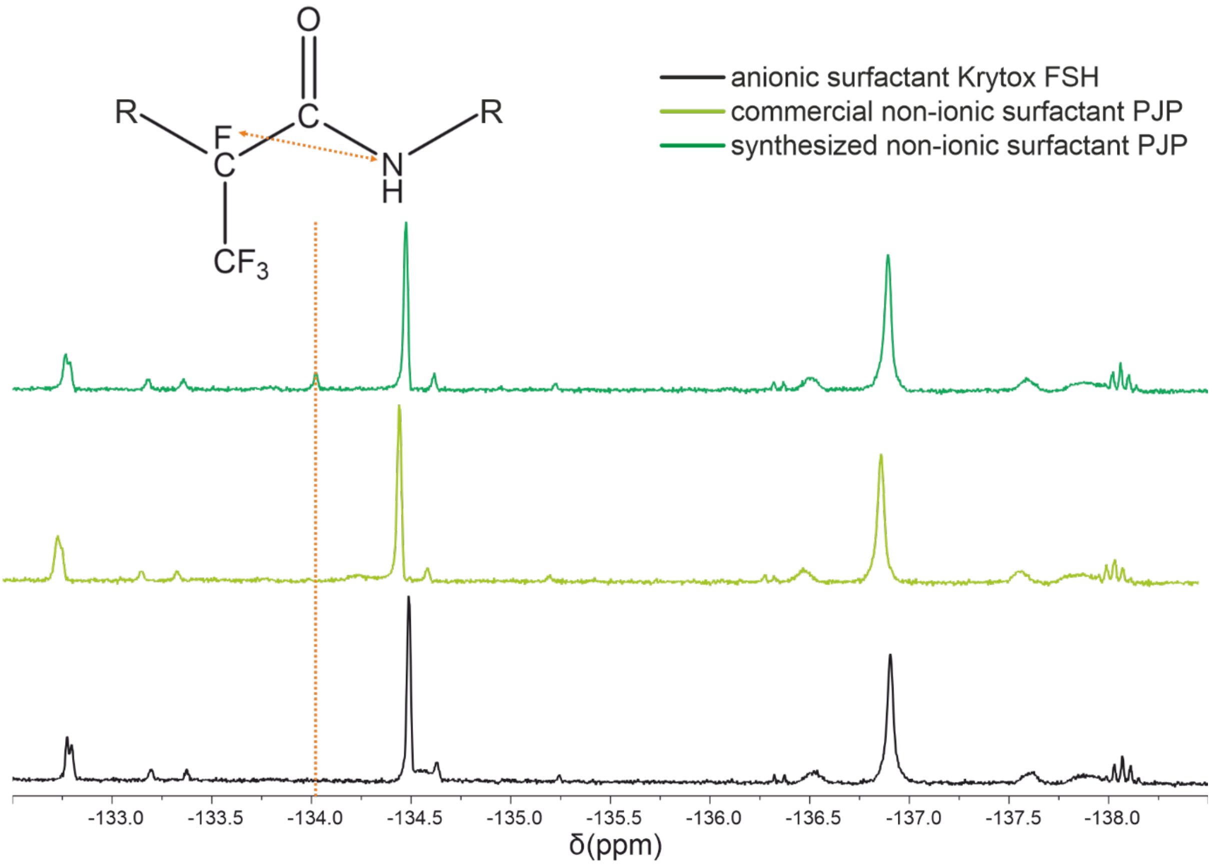

Figure S4. NMR spectra of the three different surfactants used in this study. A signal at -134.025 ppm can be observed for the synthesized PJP surfactant. We attribute this peak to coupling between the F-atom at the α-C and the N-Atom, indicated by the orange arrow. The absence of this peak in the commercially available surfactant supports our hypothesis of large amounts of anionic Krytox FSH impurities, corroborating the FT-IR investigation shown in Figure 4.

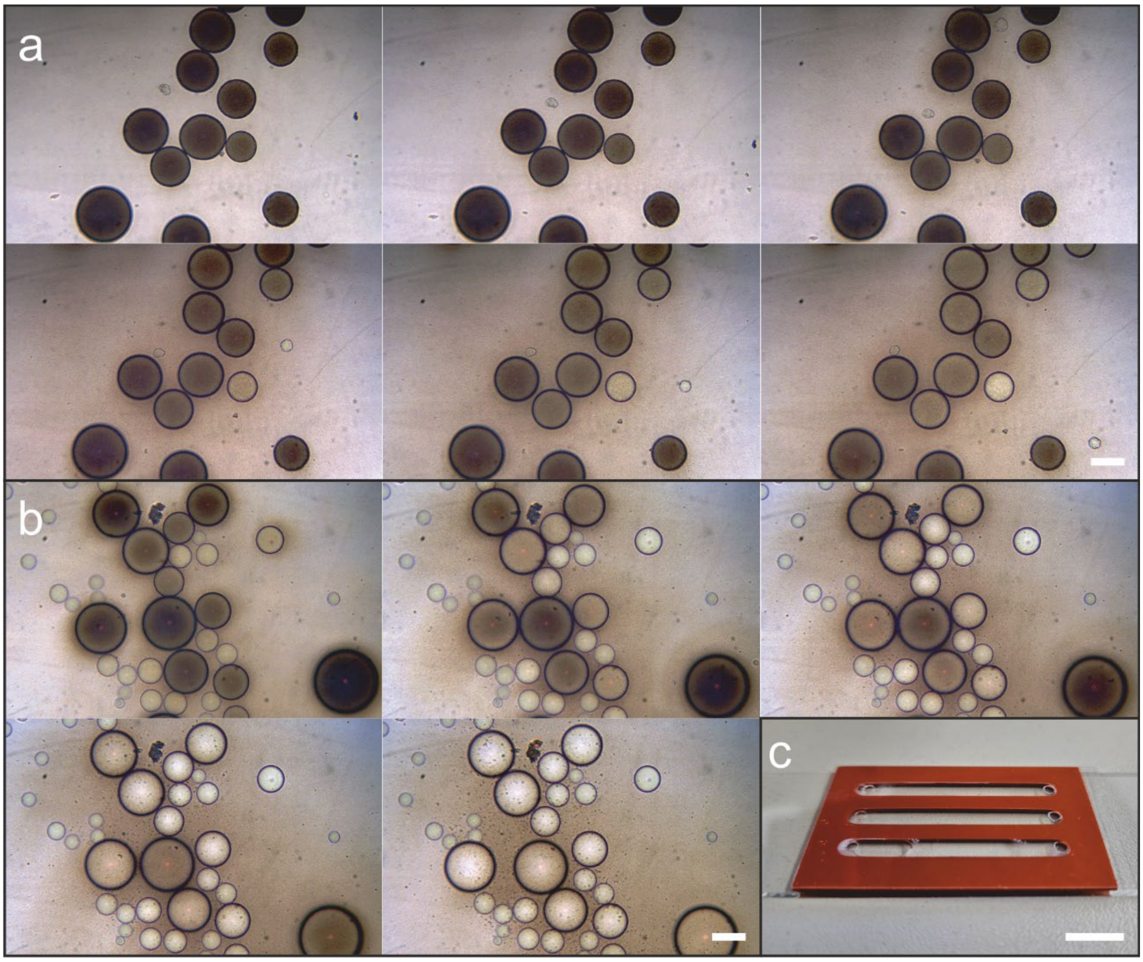

Figure S5. Light microscopy images of aqueous droplets laden with positively charged particles in fluorinated oil stabilized by non-ionic surfactants. The emulsions were stabilized with (a) 0.1 wt.-% commercial PJP or (b) 0.1 wt.-% self-synthesized PJP. In both cases, the microscopy images show particles leaving the droplets over time (left to right, top to bottom). (c) The emulsions were produced by vortexing and then transferred to a capillary shown here to avoid fast drying in air.

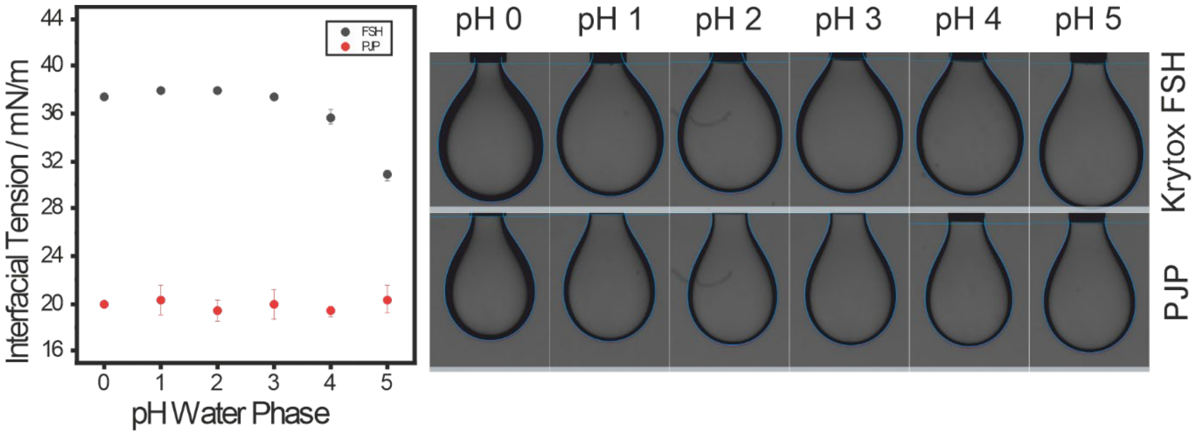

Figure S6. Interfacial tension between a droplet of fluorinated oil (Novec HFE 7500) containing 0.1 wt.-% of either Krytox FSH or PJP surfactant and water at different pH values. The interfacial tension was generally lower for the PJP surfactant and remained constant at all pH values. The anionic Krytox stabilizes the emulsion well at moderate pH values but is less interfacially active at low pH due to the protonation of the carboxylic acid headgroup.

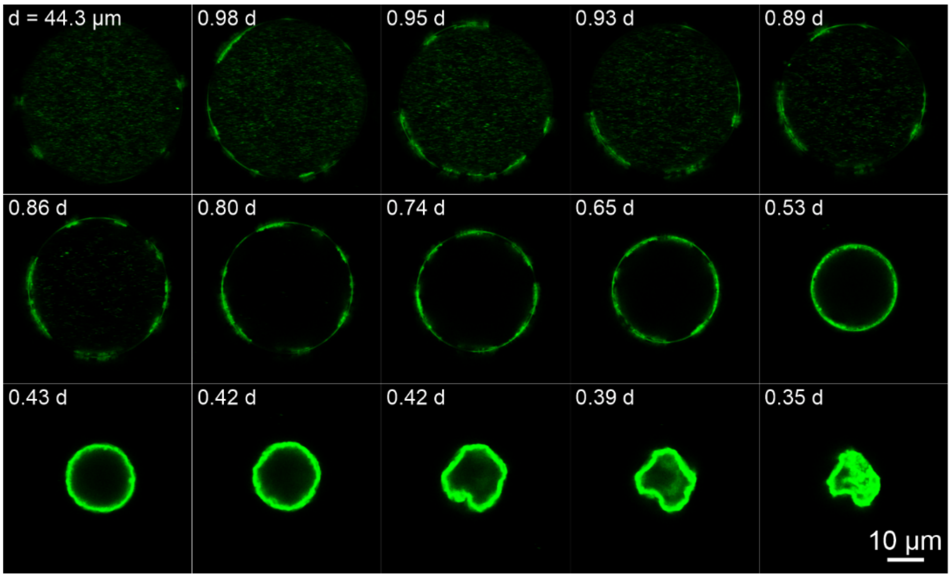

Figure S7. Confocal images of the middle plain of a particle-laden droplet (200nm, 0.27 wt.-%) with initial pH 2 at different times during the drying process. Early adsoption of the particles to the interface leads to thin-sheet buckled structures.

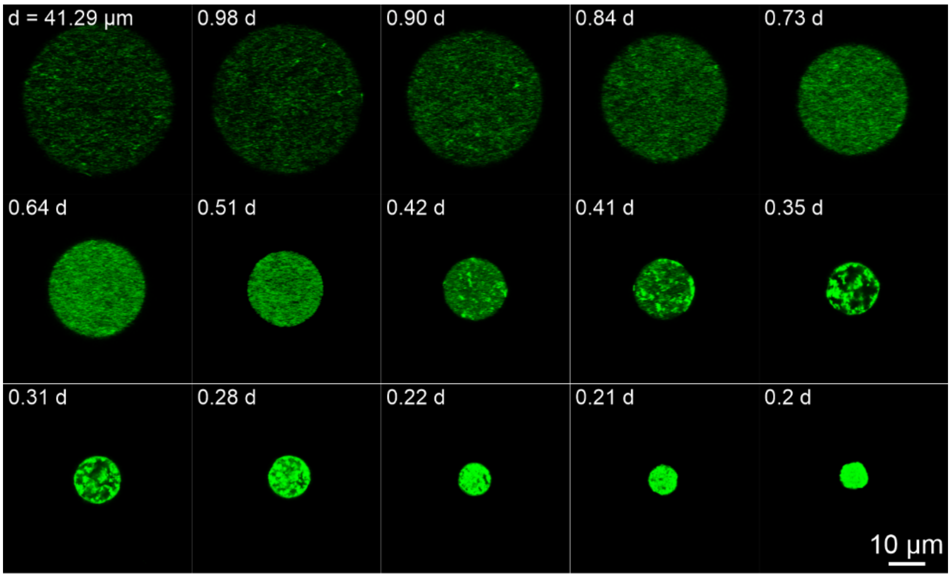

Figure S8. Confocal images of the middle plain of a particle-laden droplet (200nm, 0.27 wt.-%) with initial pH 3 at different times during the drying process. Late adsorption of the particles to the interface leads to nearly spherical supraparticles with undulated surface patterns.

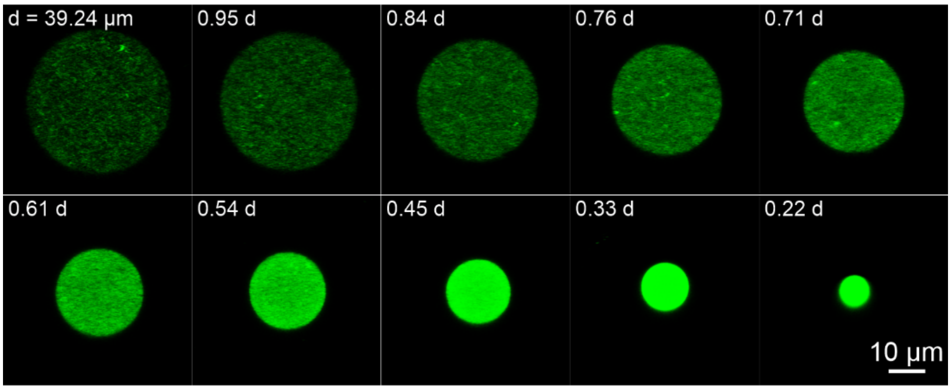

Figure S9. Confocal images of the middle plain of a particle-laden droplet (200nm, 0.27 wt.-%) with initial physiological pH at different times during the drying process. Adsorption of the particles to the interface is suppressed throughout the entire drying process and result in spherical supraparticles.

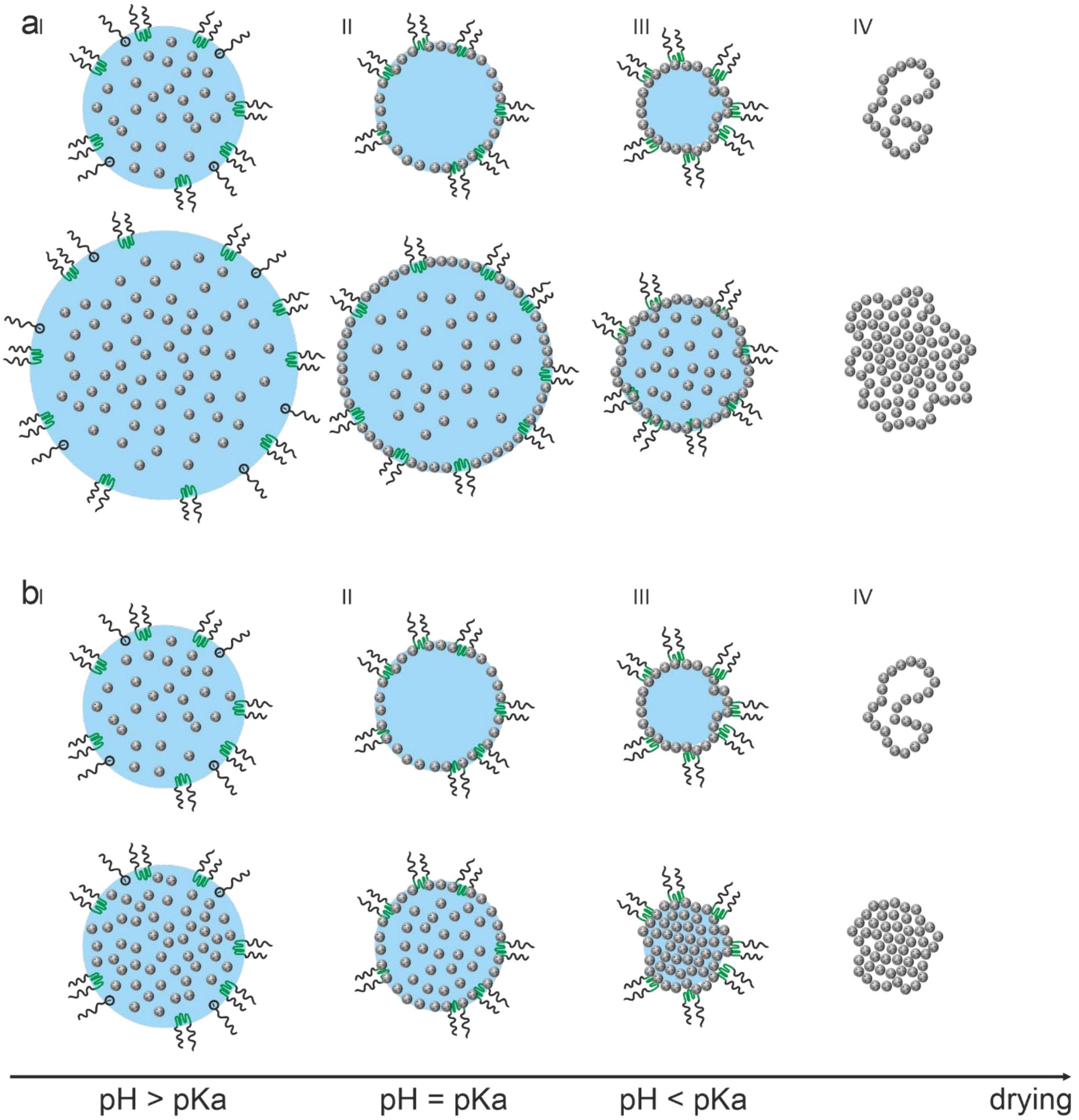

Figure S10. Schematic representation of particle-laden aqueous droplets during drying. (a) Larger droplets have a higher portion of particles left in the bulk after interfacial adsorption, resulting in less buckled structures. (b) For same size droplets, the particle concentration dictates the final morphology: A higher particle concentration leads to less buckled structures, since more particles are left in bulk after interfacial adsorption triggered by protonation of the surfactant.

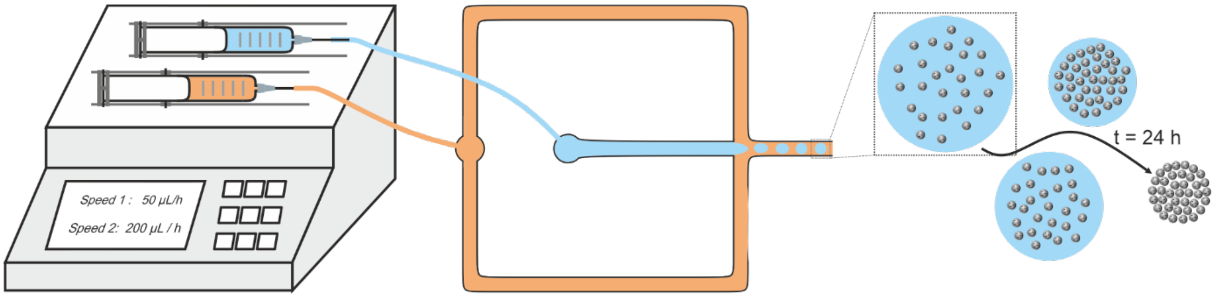

Figure S11. Schematic representation of the microfluidic setup used to make supraparticles. Both fluids are ejected from the syringes in a controlled manner using a syringe pump (left) and injected into a microfluidic channel (middle, simplified) The particle-laden water phase is injected into the inner channel while the fluorinated oil phase, which contains the surfactant, is injected into the outer channel. At the junction where both liquids meet, the water phase is sheared off and equally sized droplets are produced. The droplets (+ oil phase) are collected and stored at ambient atmosphere. The water droplets dry in approximately 24 -36 hours (right side) and supraparticles dispersed in the oil phase are obtained.

Table S1. Equations and values used for the calculations of the Péclet-number in the case of overnight drying and the case of drying during confocal imaging. Both cases describe the sample with the fastest drying observed (in their respective setups) and the largest initial droplet size. As a result, the calculated value of Pe is the maximal value that would describe the present particle systems. For the viscosity of the dispersion, the value of pure water was taken, since the initial particle concentration of 1 wt.-% affects the viscosity only negligibly.

Variables and equations[73]			Case 1: Drying over night	Case 2: Drying in confocal
Name	Variable	Unit	Value	
Péclet number	$Pe = \frac{\tau_D}{\tau_{ev}}$	-	3.5×10^{-3}	3.7×10^{-2}
Characteristic time of diffusion	$\tau_D = \frac{R_0^2}{D_0}$	s	298.09	202.03
Initial droplet radius	R_0	m	23×10^{-6}	21×10^{-6}
Stokes-Einstein diffusion coefficient	$D_0 = \frac{k_B \times T}{3 \times \pi \times \eta \times x}$	$\frac{m^2}{s}$	1.77×10^{-12}	2.18×10^{-12}
Boltzmann constant	k_B	$\frac{J}{K}$	1.38×10^{-23}	
Temperature	T	K	298.15	
Viscosity of the dispersion	η	$Pa \times s$	0.001	
Primary particle diameter	x	m	246×10^{-9}	200×10^{-9}
Characteristic time of evaporation	τ_{ev}	s	86400 (24 h)	5400 (90 min)

Table S2. Total number of supraparticles (n) evaluated for every statistic, which is shown in Figure 5 and Figure 7.

Figure	Initial pH					
5	2	2.25	2.5	2.75	3	4
n	76	80	182	70	59	52

Figure	Initial droplet size / µm			
7a	20	25	31	46
n	136	182	118	55

Figure	Initial particle concentration	
7b	1 wt.-%	5 wt.-%
n	62	132

Table S3. Measured droplet sizes and supraparticle sizes that were obtained after evaporation of these droplets (Figure 7a). The supraparticle sizes were measured using the rough, spherical supraparticles to ensure the best comparability between the samples. The linear relation can be seen by the good match of the droplet size and supraparticle size ratios (Referring to the smallest size droplet/supraparticle respectively).

Droplet size [µm]	Supraparticle size [µm]	Droplet size ratio	Supraparticle size ratio
**20**	6.11 ± 0.12	1	1
**25**	7.53 ± 0.23	1.25	1.23
**31**	9.06 ± 0.22	1.55	1.48
**46**	14.00 ± 0.4	2.3	2.29

Video S1. Consolidation of a particle laden-droplet (200nm, 0.27 wt.-%) with initial pH 2

Video S2. Consolidation of a particle laden-droplet (200nm, 0.27 wt.-%) with initial pH 3

Video S3. Consolidation of a particle laden-droplet (200nm, 0.27 wt.-%) with initial pH 5
