# Supplementary figures and images for: Control of Buckling of Colloidal Supraparticles
Source: Small. 2025 May 2;21(22):2411772. doi: 10.1002/smll.202411772 (PMC12138862; doi:10.1002/smll.202411772)

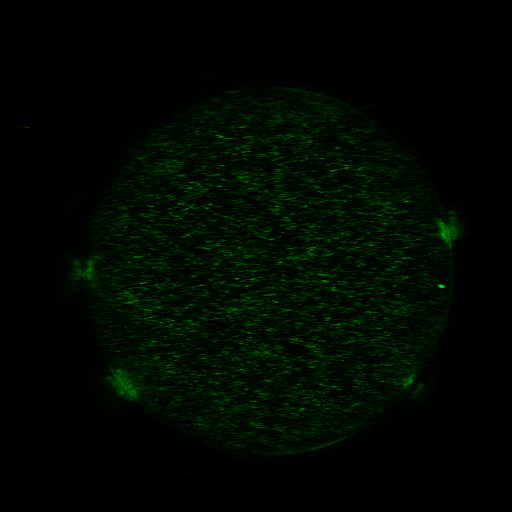

Supplement: Supplementary file 2 — Supplemental Movie 1 [file SMLL-21-2411772-s004.gif]

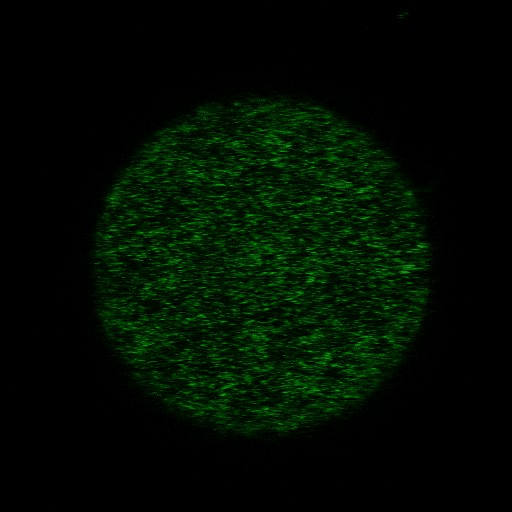

Supplement: Supplementary file 3 — Supplemental Movie 1 [file SMLL-21-2411772-s001.gif]

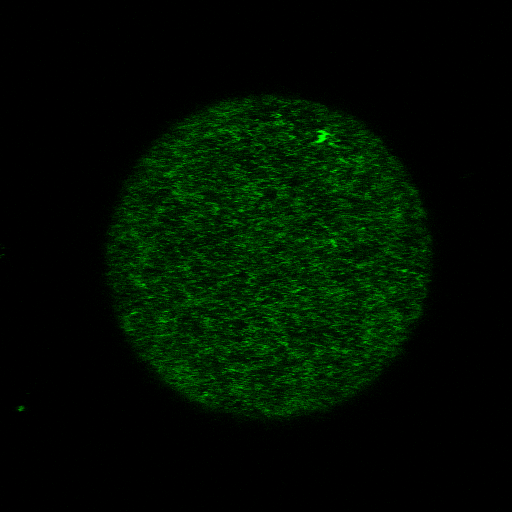

Supplement: Supplementary file 4 — Supplemental Movie 1 [file SMLL-21-2411772-s002.gif]
